# Is the Tendon-to-Groove Ratio Associated with Elevated Risk for LHB Tendon Disorders?—A New Approach of Preoperative MR-Graphic Analysis for Targeted Diagnosis of Tendinopathy of the Long Head of Biceps

**DOI:** 10.3390/jcm13102860

**Published:** 2024-05-13

**Authors:** Kristina Gerhardinger, Lisa Klute, Christian Pfeifer, Josina Straub, Laura Hechinger, Moritz Riedl, Volker Alt, Maximilian Kerschbaum, Leopold Henssler

**Affiliations:** 1 Department of Trauma Surgery, Orthopaedics and Sports Medicine, Hospital Barmherzige Brueder, 93049 Regensburg, Germany; 2 Department of Trauma Surgery, University Hospital Regensburg, 93053 Regensburg, Germany; 3 Department of Trauma and Hand Surgery, Innklinikum Altötting, 84503 Altötting, Germany

**Keywords:** shoulder, biceps tendon, intertubercular groove, arthroscopy, pulley lesions

## Abstract

**Background**: Pathologies of the long head of the biceps (LHB) tendon are a common cause of anterior shoulder pain. While the influence of the anatomical morphology of the intertubercular groove (ITG) on the development of LHB tendon instability has been investigated with ambiguous results, the relationship of the LHB to ITG anatomy has not yet been considered in this context. The objective of this study was to reliably extract the tendon-to-groove ratio from MRI scans of symptomatic patients and examine its potential influence on the occurrence of certain causes for LHB-associated symptoms. **Methods**: In this retrospective study, preoperative MRI scans of 35 patients (mean age of 46 ± 14 years) presenting with anterior shoulder pain and clinical indications of LHB tendinopathy were analyzed in transversal planes. Long and short diameters of the LHB tendon and ITG were measured, cross-sectional areas of the LHB tendon and ITG were calculated from these measurements, and the ratio of cross-sectional areas (LHB/ITG) was introduced. All measurements were repeated independently by three investigators and inter-rater reliability was assessed using intraclass correlation coefficient (ICC). Thereafter, tendon-to-groove ratios were compared in patients with and without intraoperative signs of LHB tendon instability. **Results**: All patients exhibited intraoperative signs of LHB tendinitis, with additional findings including pulley lesions and SLAP lesions. Analysis revealed variations in the dimensions of the LHB tendon and ITG cross sections, with the tendon-to-groove ratio decreasing from 37% at the pulley to 31% at the deepest point of the sulcus. Very good inter-rater reliability was observed for all measurements. The tendon-to-groove ratio did not significantly differ (*p* > 0.05) in patients with or without pulley lesions or SLAP lesions. **Conclusions**: Our study introduced the novel parameter of the tendon-to-groove ratio of cross-sectional areas as a reproducible parameter for the description of local anatomy in the field of targeted diagnosis of LHB tendon disorders. While our findings do not yet support the predictive value of the tendon-to-groove ratio, they underscore the importance of further research with larger cohorts and control groups to validate these observations.

## 1. Introduction

Pathologies of the long head of the biceps (LHB) tendon are a common cause of anterior shoulder pain [1]. In over 75% of arthroscopic surgeries of the shoulder, concomitant pathologies of the LHB tendon can be found [2], which require treatment. In addition, the number of arthroscopic biceps tenodesis procedures has constantly increased over the past decade because the field of indications has widened [3,4,5]. Common pathologies of the LHB tendon include tendinitis, tenosynovitis, subluxation, dislocation, and tears [6,7]. These conditions often manifest as anterior shoulder pain, weakness, and clicking sensations during arm movements. While tendinitis and tenosynovitis typically result from overuse or degeneration, subluxation and dislocation can occur due to instability of the biceps tendon within the intertubercular groove (ITG) [7,8,9,10]. Tears of the LHB tendon may occur acutely due to trauma or as a result of degenerative changes. These pathologies can significantly impair shoulder function and quality of life, necessitating prompt diagnosis and appropriate management strategies [5,10].

Originating from the supraglenoid tubercle and partly from the superior glenoid labrum, which is summarized as the SLAP (superior labrum from anterior to posterior) complex, the LHB tendon resides intra-articularly but extrasynovially, encompassed by a synovial-lined sheath extending into the ITG [3,6]. Proximal to the intertubercular groove the coracohumeral ligament and superior glenohumeral ligament fuse along the lateral half of the rotator interval with fibers from the subscapularis and supraspinatus tendons to form the biceps reflection pulley—a sling-like structure stabilizing the long head of the biceps tendon [11,12] at its entry to the intertubercular groove. Upon passing the biceps reflection pulley, the LHB tendon undergoes a 30- to 40-degree angulation as it enters the intertubercular groove [3,13]. The integrity of the tendon’s fixation system of the SLAP complex and biceps pulley system is crucial since instability of the LHB tendon may not only lead to painful LHB tendinitis but also can cause rotator cuff lesions [7,10,14].

From a physiological viewpoint, it is certain that the resultant force vector, that builds up during arm loading, varies—in certain glenohumeral positions, it may point directly into the intertubercular groove, stabilizing the tendon, whereas in other positions, the tendon may directly load the pulley reflection complex, potentially causing instability or injury [13]. Thus, due to LHB instability, e.g., caused by pulley lesions, the tendon gets successively damaged by moving over the tuberosities sideways during rotation of the arm [7].

At the same time, lesions of the biceps pulley system are sometimes difficult to detect on preoperative MR imaging unless there is a subluxation of the tendon. Therefore, it would be helpful for treating physicians to look for indirect signs or predisposing anatomical conditions in patients suffering from LHB-related symptoms without clear evidence of structural damage in MR imaging.

Anatomical variations in the size, depth, and configuration of the intertubercular groove may also influence force vectors and the likelihood of LHB instability [7,15,16,17,18]. Consequently, the presence of certain anatomical features reliably assessable in pre-operative MR imaging, could serve as indicators for present LHB tendon pathologies [19,20].

Different morphological configurations of the ITG have already been the subject of numerous investigations as possible predispositions for the development of an instability of the LHB tendon, coming to different results [16,21,22].

However, to the authors’ knowledge, there is no study that examines the influence of the LHB tendon’s dimensions and, in particular, its dimensions in relation to the dimensions of the intertubercular groove. To consider only the individual ITG anatomy in the individual predisposition to instability and to disregard the different dimensions of the LHB tendon represents a very one-sided and therefore incomplete view of the interaction of the two anatomical structures affected.

Therefore, the objective of this study was to examine the morphology of the ITG and long head of biceps tendon, including the cross-sectional areas of the LHB tendon and ITG and to assess whether the tendon-to-groove ratio can be reliably extracted from preoperative MRI scans by different examiners.

The second objective was to assess whether this new parameter may be valuable as a prognostic factor for structural LHB tendon disease that could aid in clinical decision making or targeted patient treatment.

## 2. Materials and Methods

### 2.1. Study Cohort

**Figure 1:**
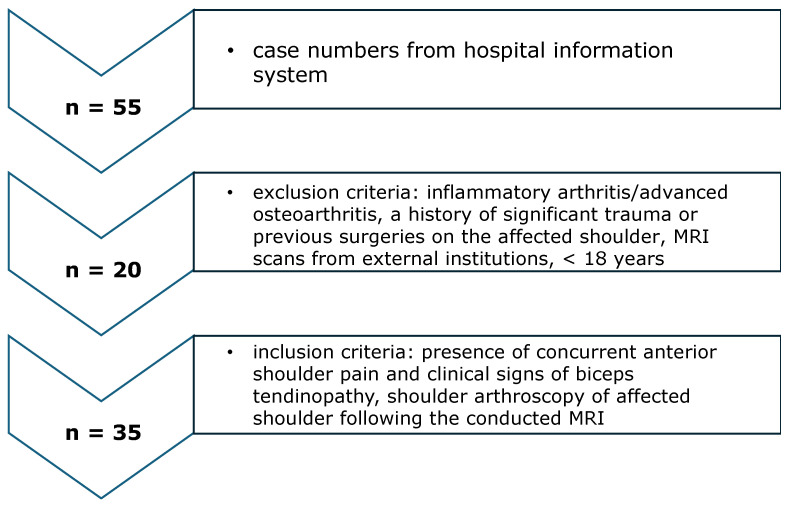
Inclusion and exclusion criteria of the study cohort.

Data were obtained from MRI scans of the shoulder region of patients who presented at our outpatient department due to acute or degenerative injuries affecting the rotator cuff or labroligamentous structures of the shoulder. Inclusion criteria encompassed the presence of concurrent anterior shoulder pain and clinical indications of biceps tendinopathy upon examination, age exceeding 18 years, and MRI scans conducted at our institution. Exclusion criteria comprised MRI scans obtained from external institutions, diagnoses of inflammatory arthritis or advanced osteoarthritis, and a history of significant trauma or previous open/arthroscopic surgeries on the affected shoulder (Figure 1). Approval from the institutional ethics review board was obtained prior to the commencement of this study (number: 18-1032-101).

### 2.2. MRI Measurements

Patients were positioned in a closed MRI scanner (MAGNETOM Skyra, Siemens Healthineers AG, Forchheim, Germany) in the supine position, with the upper extremity placed alongside the body, the elbow fully extended, and the forearm in a neutral rotation. All MRI scans were conducted utilizing the same scanner. Standard shoulder sequences were executed using a specialized shoulder coil, incorporating proton density (PD) sequences for acquiring images in the axial plane (repetition time/echo time (TR/TE) range: 3000–3220/33 ms; section thickness: 3 mm; field of view: 15 cm), which were of primary interest for this investigation. Image analysis was performed utilizing the syngo PACS viewer (Siemens Healthineers AG, Forchheim, Germany). The positioning of the long head of the biceps tendon in relation to the entire length of the intertubercular groove was evaluated on axial PD-weighted images, with measurements taken both at the entrance of the intertubercular groove (measuring point 1) and at its deepest point (measuring point 2).

**Figure 2:**
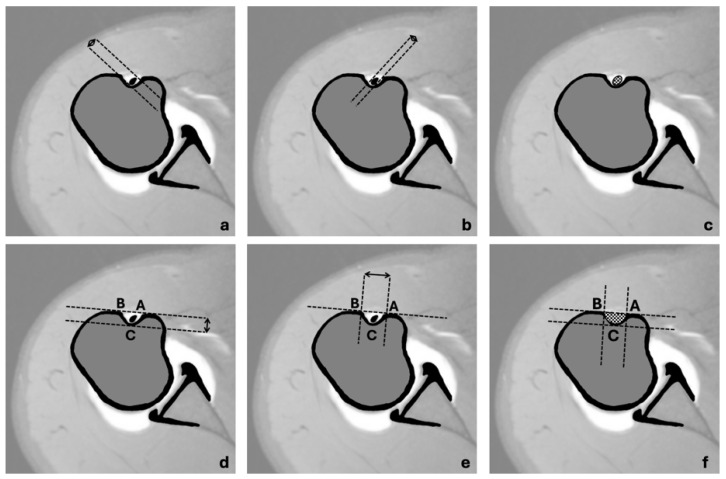
Dimensions that were extracted from axial PD images at both measuring points: Long axis of the LHB tendon (**a**) and short axis of the LHB tendon (**b**). For the measurement of the dimensions of the ITG various straight lines and points were defined: First, a straight line was created that was tangent to both edges of the ITG. The point of contact of the tangent at the lesser tuberosity was labeled A and the point of contact at the greater tuberosity was labeled B. A second parallel straight line was then drawn and shifted in parallel so that it was tangent to the deepest point of the ITB. The point of contact of the second tangent with the base of the ITG was labeled C. The depth of the ITG was measured as the distance between the two straight lines (**d**). The distance between points A and B was used to measure the width of the ITG (**e**) and depth of the ITG (**d**); the cross-sectional areas of both the LHB tendon (**c**) and the ITG (**f**) were calculated from the above measurements.

**Figure 3:**
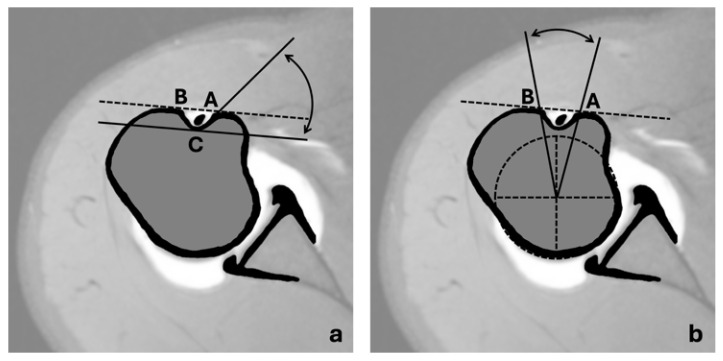
The medial wall angle (MWA, **a**) was determined by measuring the angle formed between a straight-line connecting points A and B and a second line intersecting points A and C. This measurement indicates the inclination of the medial wall of the ITG as it transitions towards the lesser tuberosity. The adapted opening angle (AOA, **b**) was assessed as an angle originating from the center of the best-fit circle of the humeral head and extending from point A to point B. This angle provides information about the degree of opening from the center of the humeral head to the indicated points.

Specific dimensions and angles were gathered for the analysis of the anatomy of the long head of the biceps tendon and the intertubercular groove in the MRI scans (Figure 2 and Figure 3):

1. Long axis of the elliptical LHB tendon cross-section (Figure 2a);
2. Short axis of the elliptical LHB tendon cross-section (Figure 2b);
3. Depth of the ITG (Figure 2d);
4. Width of the ITG (Figure 2e);
5. Medial wall angle (MWA, Figure 3a);
6. Adapted opening angle (AOA, Figure 3b).

**Figure 4:**
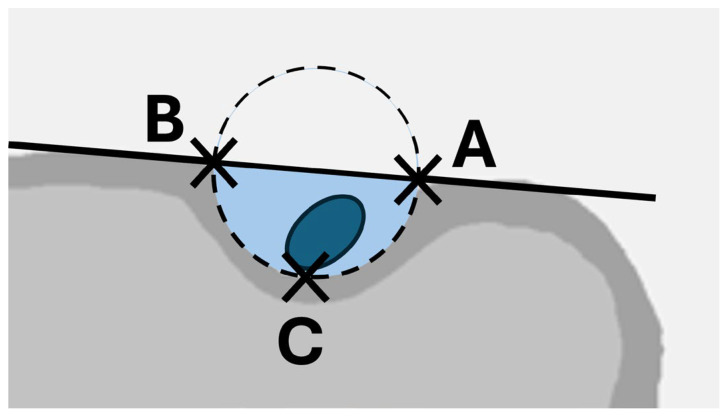
Visualization of the area ratio between the LHB tendon and the intertubercular grove. While the cross-sectional area of the LHB tendon is calculated under the assumption of an elliptic tendon shape (dark blue), the area of the groove was calculated under the assumption of a half-elliptic shape (light blue). Both cross-sectional areas (A–C) were calculated as explained in Figure 2.

The cross-sectional area of the ITG was calculated under the assumption of a semi-elliptical shape (A = π × 0.5w × d × 0.5), utilizing the measured dimensions of width (w) and depth (d) as seen in Figure 4. Similarly, the cross-sectional area of the LHB tendon was calculated (A = π × 0.5w × 0.5d) based on the gathered measurements.

Three independent observers (experienced shoulder surgeons) conducted a review of the axial PD slices from each shoulder MRI scan. The examiners were instructed to measure the long and short axis of the tendon and groove anatomy both at the entrance to the ITG and at the deepest point of the ITG. To ensure a high degree of reproducibility and comparability of the measurements, the examiners had visual instructions for the measurements to be determined shown in Figure 2 and Figure 3. All reviewers were blinded to the patients’ clinical data and conducted all measurements on their own without knowledge of the other examiners data. 

### 2.3. Statistical Methods

The normality of the data was assessed using Shapiro–Wilk testing, revealing a normal distribution for all measured parameters. However, the calculated parameters, including cross-sectional areas and ratios of cross-sectional areas, did not exhibit a normal distribution. Consequently, continuous data were analyzed and expressed as the mean and standard deviation for normally distributed data, while the median and interquartile range (IQR) were used for non-normally distributed data. Categorical data were presented as total count and percentage. Chi-squared tests were utilized for comparing categorical data, and group comparisons for continuous data were performed using the Mann–Whitney *U* Test for non-normally distributed variables and two-sided *t* test for normally distributed parameters. Inter-rater reliability was expressed with the intraclass correlation coefficient (ICC). Statistical analyses were conducted using IBM SPSS Statistics Version 29 (IBM Inc., Armonk, NY, USA), with significance set at *p* < 0.05.

## 3. Results

### 3.1. Patient Collective

We included 35 patients (30 males, 85.7%) with a mean age of 46 ± 14 years (19–73 years). All patients showed intraoperative signs of tendinitis of the LHB tendon. A total of 14 patients (40.0%) were intraoperatively diagnosed with rotator cuff tears, 5 of them with anterosuperior lesions and concomitant lesions of the biceps reflection pulley. Four additional patients (11.4%) showed isolated pulley lesions (PL) without major injury of the rotator cuff. Other intraoperative findings indicating the need for LHB tenodesis were SLAP lesions (6/35 patients, 17.1%) and partial ruptures of the intraarticular portion (5/35 patients, 14.3%).

### 3.2. Anatomy of the LHB Tendon

The long diameter of the LHB tendon (6.6 ± 2.0 mm) decreased from the entrance of the sulcus (measuring point I) to the deepest point of the sulcus (measuring point II, 6.0 ± 1.4 mm; *p* = 0.009). In contrast, the shorter diameter of the LHB tendon increased in its course and amounted to 2.2 ± 0.6 mm at the sulcus entrance and 2.7 ± 0.5 mm in the middle ITG (*p* < 0.001). Thus, the aspect ratio of the short to the long axis was calculated to a median of 31% (IQR 28–42%) at the pulley and 43% (IQR 39–52%) in the deep ITG. The cross-sectional area of the LHB tendon was calculated from the single measurements and amounted to a median of 9.8 mm^2^ (IQR 8.0–14.9 mm^2^) at the pulley complex and 12.5 mm^2^ (IQR 9.5–14.5 mm^2^) at the second measuring point at the deepest point.

All the measurements of the long axis of the tendon (ICC 0.999), short axis of the tendon (ICC 0.991), and the LHB cross-sectional area showed very good inter-rater reliability (ICC 0.995).

Summing up these results, the cross-sectional area of the LHB tendon did not undergo statistically significant changes (*p* = 0.232), even if the changes in the ratio of the short to long axis indicate a change in the shape of the LHB tendon from a relatively flat and oval shape of the intraarticular portion at the pulley complex to a significantly rounder shape of the tendon (*p* < 0.001) over the course through the bicipital groove.

The LHB tendon’s dimensions did not significantly differ in patients with and without lesions of the SLAP complex or pulley system (see Table 1).

### 3.3. Anatomy of the Intertubercular Groove

The average dimensions of the intertubercular grove were 11.0 ± 1.5 mm in width and 3.5 ± 0.8 mm in depth at the entrance to the intertubercular grove. At its deepest point, the width of the ITG did not change significantly (10.5 ± 1.9 mm; *p* = 0.081) but the depth increased to 4.9 ± 0.5 mm (*p* < 0.001).

Accordingly, the cross-sectional area of the ITG increased from a median of 29.7 mm^2^ (IQR 24.4–36.7 mm^2^) at measuring point I to a median of 39.3 mm^2^ (IQR 32.4–47.4 mm^2^) at measuring point II (*p* < 0.001). We found very good inter-rater reliability for measurements of the ITG width (ICC 0.998) and ITC depth (ICC 0.995) and cross-sectional area of the ITG (ICC 0.996), respectively.

The medial wall angle (MWA) and the adapted opening angle (AOA) measured an average of 45.5 ± 7.1° and 21.3 ± 3.7°, respectively.

Moreover, none of the above mentioned ITG dimensions and angles significantly differed in patients with and without SLAP or pulley lesions (see Table 1).

### 3.4. The LHB-Tendon-to-Intertubercular-Groove Ratio

The tendon-to-groove ratio of LHB/ITG was determined with very-high inter-rater reliability (ICC 0.991).

Since the cross-sectional area of the LHB tendon over the course from the joint through the ITG remained unchanged and, at the same time, the cross-sectional area of the ITG increased from entrance to deepest point of the ITG, the tendon-to-groove ratio decreased over the tendons course.

The median tendon-to-groove ratio of the cross-sectional areas (LHB/ITG) was significantly higher (37% [IQR 25–54%]) at the pulley complex compared to the ratio at the deepest point of the ITG (31% [IQR 22–38%]; *p* = 0.005).

Neither the ratio of cross-sectional areas nor any of the other measurements or the calculated parameters showed significant differences depending on the presence or absence of a pulley or SLAP lesion (Table 1).

## 4. Discussion

In our study, dimensions of the long head of biceps tendon and the intertubercular groove were assessed by three independent examiners using preoperative MRI in neutral arm position in patients with anterior shoulder pain and intraoperative signs of tendinitis of the LHB tendon.

Key findings of the study were as follows:

1. The tendon-to-groove ratio of cross-sectional areas was introduced as a new parameter of local anatomy and showed very-high inter-rater reliability;
2. The cross-sectional area ratio of LHB tendon to intertubercular groove decreased from 37% to 31% over its course from the biceps pulley system to the deepest point of the ITG;
3. Neither at measuring point 1 nor at measuring point 2 did the LHB/ITG ratio differ in patients with vs. without pulley lesions or SLAP lesions.

### 4.1. Intertubercular Groove Morphology and Its Influence on LHB Instability

Many previous studies have focused on certain anatomical configurations of the ITG and their effect on LHB tendon instability and thus higher likelihood of tendinopathy. The current state of knowledge of the available studies on this topic suggests that the depth or width of the groove alone do not play important roles in the pathogenesis of tendon instability but rather the morphology in its entirety. Therefore, the medial wall angle as well as the total opening angle are commonly used parameters to quantify the anatomy of the intertubercular groove. Therefore, all of these parameters were also evaluated in our study to ensure comparability to existing literature.

The depth of the intertubercular groove at its deepest point of 4.9 ± 0.5 mm (measuring point 2) in our cohort complies with results of similar studies. Abboud et al., for example, also assessed MR imaging, measuring a median depth of 5.1 ± 1.97 mm, and Shah et al. found a depth of 5.1 mm in a group of patients with LHBT pathology in comparison to a depth of 5.5 mm in a healthy control group [21,23]. Kavak et al. assessed the depth of the ITG in patients with unstable LHB tendons as well as in patients with stable LHB tendons [24]. The ones with unstable tendons showed an average depth of 5.47 ± 0.23 mm which was also concordant with the results of our study. The healthy control group in the study showed deeper grooves (5.90 ± 0.21 mm) [24]. Looking at the width of the ITG, investigating X-rays of 37 patients with anterior shoulder pain and comparing the results to those of a control group, Pfahler et al. measured a depth of 11.6 mm on average. This is also concordant with the results of the present study which measured 11.0 ± 1.5 mm at the cranial entrance to the ITG (measuring point 1) as well as at the measuring point at maximal ITG depth (measuring point 2) [22].

Contrarily, the mean MWA of 45.5 ± 7.1° measured in the present study diverges almost 10 degrees from the angle Kavak et al. published in 2019 even though they measured the MWA at the deepest point of the ITG using the same reference points as in our study [24]. Kavak et al. showed generally higher angles in patients with stable as well as unstable LHB tendons, with an MWA of 54.66 ± 0.60° and 52.16 ± 0.44°, respectively. Kavaks results resemble the ones of Ulucakoy’s study, that showed 49.9 ± 8.4° in the group with unstable LHBT and 51.0 ± 8.6° in a healthy reference group [24,25]. In contrast, the MWA that Shah or Pfahler et al. measured complied well with the ones of the present study, only differing by 2° and 3°, respectively [22,23].

All of these existing investigations are based on the physiological assumption that a deeper groove results in better stabilization of the LHB tendon due to better guidance by the bony walls of the ITG. In 2017, Yoo et al. showed that a shallow intertubercular groove, identified by a larger opening angle, smaller medial angle, and shallower depth may represent a predisposing factor for LHB tendon instability [26]. More than a decade earlier, Spritzer et al. already postulated a strong correlation between ITG morphology and LHB tendon instabilities [27], limited by a small group size and lack of statistical analysis. Taking a closer look at LHB tendon instability and possible subsequent pathologies, Kleim et al. analyzed different types of biceps reflection pulley (BRP) lesions on the basis of arthroscopy findings and showed that lateral BRP injuries are not influenced by the ITG morphology, whilst medial and bilateral ones are [28]. They presumed that a shallow BG morphology may lead to a dislocation of the LHBT, which may move out of the BG medially or laterally while external or internal rotation movement of the humerus subsequently damages the medial or lateral BRP [28]. Looking at this “Skipping” of the LHB tendon out of the ITG, while rubbing over its rims from a pathophysiological viewpoint, it seems at least possible that the LHB tendon itself has a high probability of getting altered or inflamed the more often this dislocation occurs. This hypothesis aligns well with Lafosse et al.’s finding that lesions of the LHB tendon occur significantly more frequently in patients with unstable tendons [7]. In contrast to all the above findings, Abboud et al. did not find any correlation between the medial wall angle or depth of the intertubercular groove and the presence of biceps tendon pathologies during arthroscopy in a study involving 75 patients, conducted with the intention to explore the relationship between ITG morphology and LHB tendon pathologies [21]. Furthermore, Ulucakoy et al. showed that a control group with stable LHB tendons had shallower ITGs with bigger opening angles than the other group with proven LHB tendon instability, concluding that the morphology of the ITG is not related to LHB tendon stability [25].

In summary, the literature on the relationship between the anatomical variants of the ITG and the occurrence of LHB instability is inconclusive. However, it is striking that all of the above-mentioned studies only examine the morphology of the groove, but none of the above studies took the influence of LHB tendon dimensions into account.

Since the dimensions of the tendon can be subject to significant changes over the course of a lifetime due to irritation, associated edematous swelling, paratendinitis, or chronic mucoid degeneration, the authors believe that a one-sided view of a bilateral problem of instability is too imprecise.

The aim of this present study was, therefore, to investigate the dimensions of both the LHB tendon and the ITG and introduce a parameter that represents the relationship between these two dimensions. For this purpose, the ratio of the cross-sectional areas of tendon-to-groove seemed to be a promising parameter, since it is conceivable that a relatively large tendon in a relatively shallow sulcus could have a higher probability of dislocation. With a corresponding injury mechanism, in which the force vector is acting on the tendon points medially, such an anatomical combination could predispose the development of a medial subluxation.

### 4.2. Cross-Sectional Areas and LHB/ITG Ratio

The presented study is the first one to assess cross-sectional areas of both the LHB tendon as well as the intertubercular groove in patients with proven LHB tendinitis. To our knowledge, the literature on these anatomical dimensions at different measuring points is very limited.

There is just one study that can be used as a reference for the cross-sectional area of the ITG: Cardoso et al. determined the cross-sectional area at the point of the ITG with maximum depth, which is equivalent to our measuring point 2. Regarding the cohort of 37 patients with LHB tendon pathology, the cross-sectional area of the ITG was 19.1 ± 7.1 mm^2^. The healthy control group showed a cross-sectional area of 16.6 ± 4.5 mm^2^ [29]. The authors summarized that even though the cross-sectional area width and depth of the ITG were gradually higher in patients with LHB tendon pathology, their study did not support any correlation between LHB tendon pathology and ITG morphology [29].

The results of both groups differ a lot from the cross-sectional area of 29.7 mm^2^ that we calculated at the equivalent measuring point. Reasons for this difference of more than 10 mm^2^ could either be that the measured dimensions of the groove included in the calculation differ or that Cardoso et al. used a different approximation formula for calculating the cross-sectional area. Either way, even small differences in the variables involved have a major impact on the result, as the formula contains an exponentiation.

Moreover, for the abovementioned rationale, the present study did not only assess the cross-sectional areas of the intertubercular groove and the LHB tendon but put both to a relation. However, no difference of the new parameter could be found in patients with or without structural lesions of the LHB tendon’s fixation points.

Even if further studies may be able to show a predictive value of the LHB/ITG ratio for present pathological alterations of the LHB, it has to be defined which clinical implications can be drawn from it. It would be conceivable that this factor could influence the decision to perform or not to perform shoulder surgery in cases where the indication is ambiguous because no clear structural lesion can be evidenced in MR images, even if the patient complains of biceps-related symptoms.

In addition, in patients who are to undergo shoulder surgery due to an existing pathology, such as a rotator cuff lesion, the likelihood of a necessary tenotomy or tenodesis of the long head of the biceps tendon could be better assessed preoperatively if the ratio suggests a pathologically altered LHBT.

### 4.3. Limitations

The present study is subject to several limitations that must be considered when evaluating the study results. First, this study is subject to a potential selection bias, since only patients with anterior shoulder pain were included in the analysis, and this study is, therefore, missing a control group without LHB tendon pathology. By adding a control group we could have investigated the differences between patients with anterior shoulder pain and healthy asymptomatic individuals. Nevertheless, the aim of the study was to investigate whether the tendon-to-groove ratio can be collected as a reliable parameter and whether the tendon-to-groove ratio can be helpful as a predictive factor for structural LHB tendon disorders in a tendinopathy cohort with suspected pulley or SLAP lesions but without clear MR graphic evidence.

Secondly, concerning radiographic imaging, only non-contrast MRI and no MR arthrography examinations were included, limiting sensitivity for the detection of SLAP lesions in preoperative scans, potentially impacting the accuracy of the findings. Nevertheless, all patients underwent arthroscopy of the shoulder, which can be considered the diagnostic gold standard for the detection of SLAP lesions and pulley lesions, and the diagnosis of SLAP lesions or pulley lesions was derived solely from the subsequent diagnostic arthroscopy. Therefore, the diagnostic accuracy of the diagnoses within this study can be considered high.

Thirdly, although the interpretation shows a good, or very good, intra- or inter-rater reliability, a certain degree of subjectivity in rating cannot be precluded.

Lastly, since our retrospective study was carried out in a small study population in a preoperativse setting, small differences between the cohorts could have been underestimated. Furthermore, a study population of only 35 patients limits the generalizability of the findings and increases the risk of selection bias. As the predictive value of the LHB/ITG ratio remained uncertain in the present possibly underpowered study, larger studies including a control cohort of asymptomatic patients without LHB tendon disorders have to clarify whether this extended preoperative analysis using the size ratio of the LHB tendon and intertubercular groove has an impact on the predisposition to LHB tendon pathologies.

## 5. Conclusions

In the pathogenesis of LHB tendon instability, a shallow morphology of the intertubercular groove has been identified as a potential predisposing factor. However, this study is the first to also consider the dimensions of the LHB tendon as a potential influencing factor and introduce the tendon-to-groove ratio of cross-sectional areas as a novel parameter in describing individual local anatomy. While this new parameter demonstrated high inter-rater reliability in this study, no significant differences were observed that could suggest the presence of LHB instability. However, this study was conducted on a cohort of patients with LHB-related anterior shoulder pain, which limits the generalizability of the findings and introduces potential selection bias. Given that this study primarily aimed to introduce the new parameter and demonstrate its reproducible assessment in a relatively small cohort without a healthy control group, further prospective investigations involving a larger study population and a healthy asymptomatic control group are necessary to explore the influence of the size ratio on the pathogenesis of LHB tendon instability and its clinical utility as a predictive factor in targeted preoperative MRI diagnostics. If subsequent studies establish a correlation between the LHB/ITG ratio and a higher likelihood of LHB tendon instability or lesions, preoperative MRI analysis of the LHB and ITG could serve as a diagnostic tool for guiding treatment decisions or predicting outcomes.

## Figures and Tables

**Table 1:** Measures and calculated parameters depending on the presence or absence of pulley lesions or SLAP lesions. LHB = long head of biceps; ITG = intertubercular groove; MWA = medial wall angle; and AOA = adapted opening angle.

	Pulley Lesion			SLAP Lesion		
	No (n = 26)	Yes (n = 9)	*p*	No (n = 29)	Yes (n = 6)	*p*
**Measuring point 1 (pulley complex)**						
Long tendon diameter (mm)	6.5 ± 1.8	7.0 ± 2.5	0.546 *	6.7 ± 2.1	6.1 ± 1.6	0.520 *
Short tendon diameter (mm)	2.2 ± 0.6	2.2 ± 0.6	0.891 *	2.2 ± 0.6	2.2 ± 0.6	0.793 *
LHB Ratio short diameter/ long diameter (%)	30 (IQR 28–42)	35 (IQR 30–40)	0.590 **	31 (IQR 28–42)	30 (IQR 28–35)	0.782 **
Cross-sectional area LHB (mm^2^)	9.6 (IQR 8.2–15.6)	11.3 (IQR 8.0–11.8)	0.985 **	10.2 (IQR 8.2–14.3)	9.5 (IQR 8.0–14.9)	0.685 **
ITG width (mm)	11.0 ± 1.5	11.0 ± 1.5	0.961 *	11.2 ± 1.5	10.4 ± 0.9	0.267 *
ITG depth (mm)	3.5 ± 0.8	3.3 ± 0.7	0.456 *	3.4 ± 0.8	4.0 ± 0.8	0.078 *
Cross-sectional area ITG (mm^2^)	28.8 (IQR 25.2–36.7)	31.7 (IQR 24.3–35.4)	0.725 **	27.9 (IQR 24.4–35.4)	33.7 (IQR 29.7–39.5)	0.272 **
Ratio LHB/ITG (%)	37 (IQR 25–49)	37 (IQR 31–54)	0.725 **	31 (IQR 28–42)	30 (IQR 28–35)	0.454 **
**Measuring point 2 (deepest ITG)**						
Long tendon diameter (mm)	5.9 ± 1.4	6.1 ± 1.7	0.817 *	5.9 ± 1.5	6.1 ± 0.8	0.751 *
Short tendon diameter (mm)	2.5 ± 0.5	2.7 ± 0.4	0.464 *	2.6 ± 0.6	2.5 ± 0.4	0.712 *
LHB Ratio short diameter/ long diameter (%)	42 (IQR 38–52)	45 (IQR 41–49)	0.697 **	45 (IQR 40–52)	39 (IQR 38–42)	0.218 **
Cross-sectional area LHB (mm^2^)	11.7 (IQR 9.0–14.5)	12.8 (IQR 10.2–13.2)	0.540 **	12.5 (IQR 9.7–14.5)	11.9 (IQR 9.5–13.1)	0.983 **
ITG width (mm)	10.4 ± 1.6	10.8 ± 2.7	0.651 *	10.7 ± 1.9	9.4 ± 1.7	0.106 *
ITG depth (mm)	4.9 ± 0.5	4.8 ± 0.5	0.609 *	4.9 ± 0.5	5.1 ± 0.7	0.260 *
Cross-sectional area ITG (mm^2^)	39.1 (IQR 32.2–47.4)	42.4 (IQR 35.7–46.1)	0.540 **	41.4 (IQR 32.2–47.4)	35.9 (IQR 35.7–41.9)	0.983 **
Ratio LHB/ITG (%)	33 (IQR 23–38)	28 (IQR 22–38)	0.985 **	30 (IQR 22–38)	33 (IQR 25–35)	0.815 **
MWA (°)	44.8 ± 6.1	47.5 ± 9.7	0.443 *	44.8 ± 6.7	48.9 ± 8.6	0.201 *
AOA (°)	21.2 ± 3.5	21.6 ± 4.6	0.833 *	21.8 ± 3.9	19.1 ± 2.0	0.117 *

* = calculated using independent *t* test; ** = calculated using Mann–Whitney *U* test.

## Data Availability

The raw data supporting the conclusions of this article will be made available by the authors on reasonable request.
